# Signed reward prediction errors drive declarative learning

**DOI:** 10.1371/journal.pone.0189212

**Published:** 2018-01-02

**Authors:** Esther De Loof, Kate Ergo, Lien Naert, Clio Janssens, Durk Talsma, Filip Van Opstal, Tom Verguts

**Affiliations:** 1 Department of Experimental Psychology, Ghent University, Henri Dunantlaan 2, Ghent, Belgium; 2 Department of Psychology, University of Amsterdam, Nieuwe Achtergracht 129-B, Amsterdam, Netherlands; Waseda University, JAPAN

## Abstract

Reward prediction errors (RPEs) are thought to drive learning. This has been established in procedural learning (e.g., classical and operant conditioning). However, empirical evidence on whether RPEs drive declarative learning–a quintessentially human form of learning–remains surprisingly absent. We therefore coupled RPEs to the acquisition of Dutch-Swahili word pairs in a declarative learning paradigm. Signed RPEs (SRPEs; “better-than-expected” signals) during declarative learning improved recognition in a follow-up test, with increasingly positive RPEs leading to better recognition. In addition, classic declarative memory mechanisms such as time-on-task failed to explain recognition performance. The beneficial effect of SRPEs on recognition was subsequently affirmed in a replication study with visual stimuli.

## Introduction

Declarative and procedural learning are key assets of the human brain. Ever since Thorndike [1], it has been thought that reward is crucial for both forms of learning. Additionally, inspired by the phenomenon of blocking [2], Rescorla and Wagner [3] proposed and modeled the concept that reward prediction is crucial for learning, and that learning occurs mainly for unexpected reward outcomes (i.e., reward prediction errors, RPEs). Their classic model foreshadowed many decades of work to come in the conditioning literature [4,5]. A recent surge of interest in this concept results from the remarkable synergy between dopaminergic recordings in the mammal brainstem (i.e., the neural signature of RPEs [6]) and the temporal-difference RPE model [7,8]. Similar views on the role of RPEs in learning were developed in other prominent theoretical frameworks (e.g., predictive coding [9] or the neoHebbian account [10]). In the ensuing empirical research, the effect of RPEs has been amply demonstrated in procedural learning paradigms such as classical and operant conditioning (e.g. [11]). However, in these procedural learning paradigms, RPEs gradually shape the acquisition of stimulus-response contingencies over multiple encounters. This is distinct from the typically human ability to learn (verbal, stimulus-stimulus) information through a single encounter by declarative learning.

Reward clearly plays a role in declarative learning [12,13] and there has been a recent surge of interest in the influence of reward on declarative memory [12,14,15]. However, strikingly, to date there is no direct empirical evidence for the behavioral impact (beneficial or otherwise) of RPEs on the acquisition of declarative information. Nevertheless, findings from procedural learning and animal research provide clear predictions on how RPEs might influence declarative learning. According to the neoHebbian learning framework [10], dopamine bursts generated by the ventral tegmental area (VTA) and projected to the hippocampus amplify long term potentiation (LTP), resulting in better memory. Rodent research has indeed demonstrated that dopamine bursts enhance learning of spatial information, even through a single encounter [16]. Given that dopamine is thought to implement RPEs [7,17,18], these findings suggest that RPEs can enhance declarative learning.

**Fig 1:**
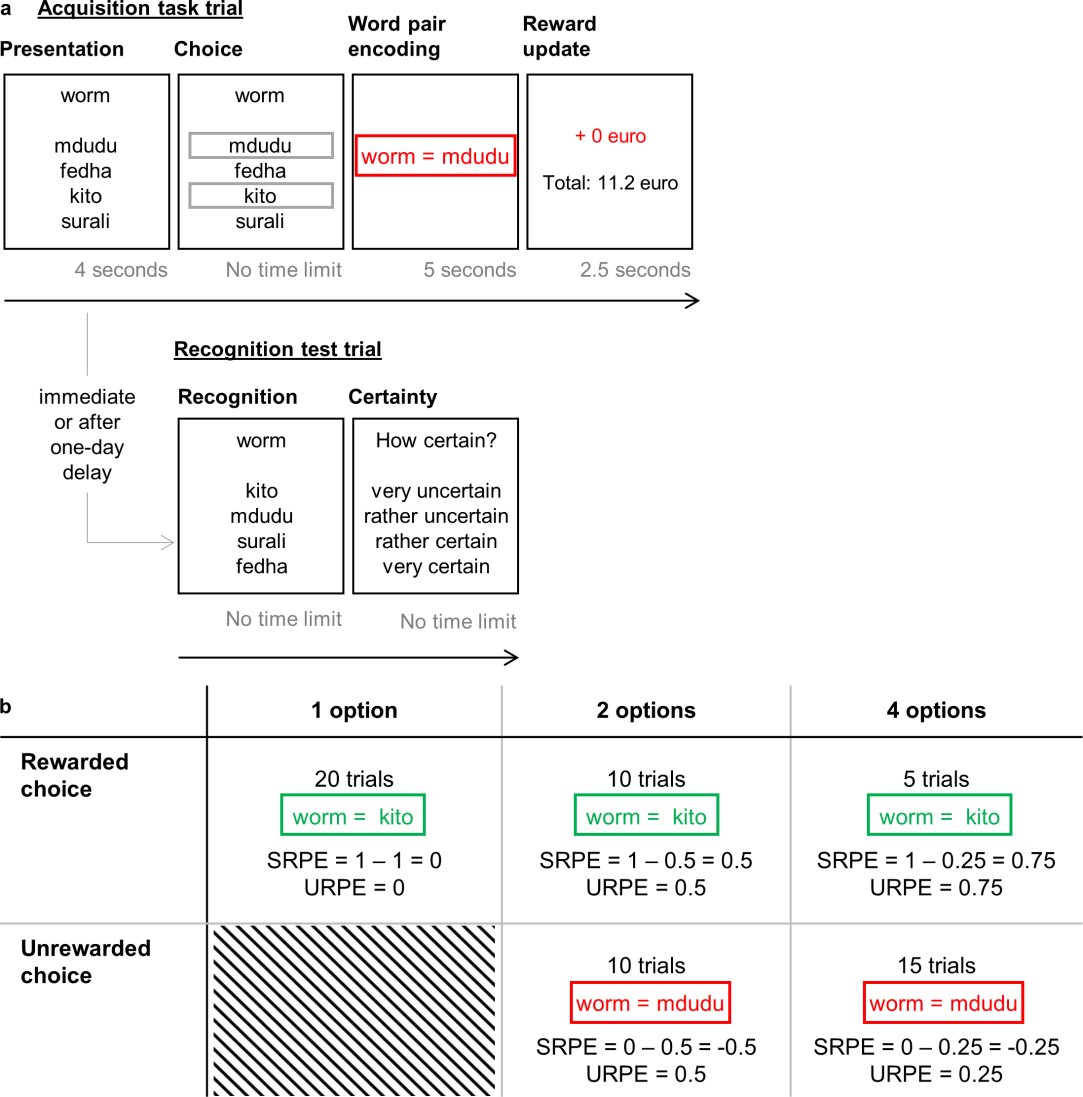
**Experiment overview (a) and experimental design (b) for Experiment 1.** (a) Participants chose between one, two or four Swahili translations in the acquisition task; the two-option condition with unrewarded choice is illustrated. Recognition and certainty were probed immediately or after a one-day delay. (b) The 3 (number of options) × 2 (obtained reward) experimental design, including number of trials and associated signed and unsigned RPE (SRPE and URPE). SRPEs were calculated by subtracting probability of reward from obtained reward; URPE is the absolute value of SRPE. The feedback is illustrated assuming that the participant chose ‘kito’ as the translation for ‘worm’.

To test the hypothesis that RPEs can indeed enhance declarative learning, we examined the impact of RPEs on declarative learning in a Dutch-Swahili vocabulary acquisition task (Experiment 1). On each trial, we presented a Dutch word accompanied by one, two or four possible Swahili translations (options) to choose from. By varying the number of available options, we manipulated the reward probability and hence the reward prediction (error). In this way, during feedback, positive and negative RPEs of known and various sizes were coupled to the valid Dutch-Swahili word pairs (see Fig 1); allowing us to empirically test whether RPEs drive declarative learning. By differentiating between positive and negative RPEs we assessed whether word pair acquisition was boosted by unsigned RPEs (URPE; indicating merely that the outcome is different than expected) or by signed RPEs (SRPE; indicating whether the outcome is better or worse than expected). Also, to test the durability of the influence of RPEs on declarative learning over time, we probed recognition either immediately or after a one-day delay. Next, we performed a first validation test on our findings by examining whether the classic time-on-task account could alternatively explain our results. As a second validation test, we performed a replication study with visual stimuli (Experiment 2).

## Experiment 1

### Methods

#### Participants

Forty participants (all university students; 32 female) enrolled in the study and were rewarded €10 for participation. Half of the participants were randomly assigned to perform the recognition test immediately after the acquisition task; the other half performed the recognition test one day later. One gift voucher of €20 was awarded to the participant with the best performance on the immediate recognition test; a second voucher was given to the participant with the best performance on the recognition test one day later. All participants were naive to the purpose of the experiment, had no prior knowledge of Swahili and had not previously taken part in any experiment involving Swahili words. Prior to the experiment all participants gave their informed consent in accordance with the Code of Ethics of the World Medical Association (Declaration of Helsinki) and were debriefed afterwards. The study has been approved by the Ethics Committee of the Faculty of Psychology and Educational Science at Ghent University.

#### Material

The experiment was run on an Asus 1215N netbook running Eprime software [19]. For the declarative learning task, 60 Dutch and 240 Swahili words were selected (see Tables 1 and 2). At the start of the experiment, participants were informed about the three parts of the study: the familiarization task, the acquisition task and the recognition test (see below for a detailed description of each part).

**Table 1:** Stimulus material: 240 Swahili words.

adhabu	chupi	jeraha	kioo	maisha	msitu	nyundo	surali
adui	daima	jibini	kisiwa	maji	msumari	nyundu	takatak
afya	dakika	jikoni	kisu	mali	mtawa	nzuri	tamasha
aibu	daraja	jiwe	kitanda	mamba	mtirka	ofisi	tanuri
akili	dari	jokofu	kitande	mapafu	mundamo	osha	tembo
alizeti	dizeli	jua	kiti	mashua	mungu	panya	trekta
amani	duka	jumatu	kito	matumai	mvringo	petye	tumbili
asili	elfu	juuya	kitovu	matumbo	mvua	picha	tumbo
baadaye	farasi	kaburi	kofia	maua	mvuke	pombe	twai
bafuni	fedha	kahawa	kovuli	mazishi	mwanake	punda	uadui
bahari	filimbi	kalamu	kuacha	mbolea	mwanga	punguza	uchorai
baharia	funzi	kamba	kuandika	mbuzi	mwezi	pwani	ufagio
baiski	furaha	kamwe	kubale	mbwa	mzungu	rafiki	ugomvi
bandari	garisi	kartasi	kubwa	mchanga	nanga	rangi	uhuru
barua	geza	katika	kudhibi	mchawi	nchi	rombus	ukame
basi	godoro	kawaida	kuhesa	mchuzi	ndaniya	sabuni	ukweli
bega	goti	kazi	kujenga	mdudu	ndege	sahani	umasijo
bendi	gundi	kelele	kukimba	mechezo	ndevu	samaki	uongo
bilaska	guruwe	kemia	kumba	mekno	ndizi	sayari	usiku
bloke	haki	kengele	kumbuka	mfuko	ndogo	seesaw	uyoga
buli	hamsi	kesho	kununa	mgonjwa	ndoora	sehemu	viatu
bunifu	hasira	kiatu	kunywa	miaka	ndugu	seri	wakala
bustani	hatua	kichwa	kupanda	mkasi	neyemba	shimoni	washia
chaki	hazini	kidole	kusanya	mkate	ngazi	shule	welder
chombo	hofu	kifua	kushoto	mkoba	ngono	simu	wengine
choori	ijayo	kihozi	kusikiza	mkuu	ngozi	singizi	wimbo
chubani	imani	kijiko	kuzama	mlango	nopya	soko	wingi
chuki	ishara	kikapu	kweli	moyo	nyange	starehe	wingu
chuma	ishiri	kimysa	leso	mpishi	nyeusi	stork	yatima
chupa	jansa	kinywa	mageho	mraba	nyota	sufuria	zeituni

**Table 2:** Stimulus material: 60 Dutch words.

agent	bord	ezel	kaas	mest	rijst	stoel	wolk
anker	brief	fiets	kassa	nacht	schat	stoom	wonde
appel	bril	goud	knie	neus	sjaal	stuur	worm
bezem	broek	graf	laken	olijf	slaap	touw	zomer
bier	brood	hamer	lamp	oven	slang	trein	
bloem	doos	haven	lepel	paard	slot	tuin	
boer	eend	hond	lijm	poort	stier	verf	
boot	emmer	hoofd	melk	regen	stift	water	

#### Familiarization task

In order to familiarize the participants with the stimuli at the start of the experiment, all Dutch and Swahili words were presented in random order for two seconds. Participants read the words in silence and pushed a keyboard button when a Dutch word appeared.

#### Acquisition task

At the start of the acquisition task, participants were informed that they were about to learn 60 Dutch-Swahili word pairs while gaining at least €8 and possibly more than €10. In addition, they were reminded of the recognition test that would follow the experiment and of the additional gift voucher of €20 for the participant with the best recognition performance.

At the start of each trial, one Dutch word was presented at the top of the screen with four Swahili words below (Fig 1A). All words remained on screen for four seconds as participants read through the options. Next, a frame appeared around the possible Swahili translations for the Dutch word. In the one-option condition only one Swahili word was framed, immediately indicating the correct Swahili translation. In the two-option condition a frame appeared around two Swahili words so participants had a 50% chance of choosing the correct translation. Finally, in the four-option condition all four Swahili words were framed, resulting in a 25% chance of choosing the correct Swahili translation. Four keyboard buttons were assigned to the four word positions and participants responded with the index and middle finger of their left and right hand. There was no time constraint on the decision but participants were encouraged to follow their first impression.

Unbeknownst to the participants, the accuracy of the chosen translations in the acquisition task was determined in advance. Specifically, a fixed number of trials was predetermined to have one, two or four valid Swahili options; and to be rewarded or unrewarded (Fig 1B). In this way, participants did not necessarily learn the actual Swahili translations of the Dutch words. For example, if a trial had been predetermined to be a two-option trial with a rewarded answer, the participants would be rewarded irrespective of their choice and this chosen word would be the translation they had to memorize. Moreover, for each Dutch word four randomly drawn Swahili words were presented, usually not including the actual translation. This made sure we had a fixed number of trials in each cell of the design (Fig 1B); moreover, it excluded any linguistic regularity in Dutch-Swahili word pairs that could influence learning. Participants were debriefed about this manipulation at the end of the experiment.

Thus, after the participants chose a Swahili translation among the possible options, feedback on the rewarded translation was given. The Dutch word, an equation sign and the (so-called) correct Swahili word appeared at the center of the screen. If the chosen Swahili translation was rewarded, a green frame was presented around the Dutch word and the chosen Swahili word, while participants heard the sound of money tumbling in a cup (three seconds). Alternatively, if the chosen Swahili translation was unrewarded, a red frame appeared around the Dutch word and one of the other possible Swahili word options, while an error buzz was played (three seconds). The words remained on the screen for five seconds and participants were instructed to use this time to learn the word pair by heart for the recognition test. The trial ended with a 2.5 seconds presentation of the total reward collected thus far. Participants won €0.28 on rewarded trials; no money was added on unrewarded trials. Because all participants were rewarded on 35 trials (see Fig 1B) the total reward always equaled €9.80, which was rounded to €10.

#### Recognition test

A magnitude comparison task was used as a filler task to reduce recency effects in the immediate recognition test. In order to keep both versions of the experiment as similar as possible, the filler task was also presented to participants who would perform the recognition test one day later. Participants categorized 400 numbers between 1 and 9 (excluding 5) as being smaller or larger than 5 (left and right button presses respectively).

At the start of the recognition test, participants were reminded about the additional gift voucher of €20 for the best-performing participant. The Dutch word appeared at the top of the screen with the same four Swahili words below. However, the order of the four Swahili words was randomized and participants were warned about this change. As soon as the words appeared, participants could choose between the four Swahili words by using the same four response buttons as in the acquisition task. No time constraints were imposed on their answer. After a Swahili word was chosen, participants indicated how certain they were about their answer: ‘very uncertain’, ‘rather uncertain’, ‘rather certain’ or ‘very certain’ (measured on a scale from 1 ‘very uncertain’ to 4 ‘very certain’). No feedback was provided after a recognition trial.

#### Data analysis

The SRPEs were calculated by subtracting the reward probability (i.e., 1, 0.5 and 0.25 probability of a rewarded choice in the one-, two- and four-option condition, respectively) from the obtained reward (i.e., 1 reward on rewarded trials and 0 reward on unrewarded trials). Thus a unique SRPE ranging from -0.50 to 0.75 was calculated for each cell in the design (see Fig 1B for a full overview). The URPEs were calculated by taking the absolute value of the SRPEs. Note that the URPEs and SRPEs differ on the unrewarded trials, allowing us to differentiate between both accounts.

Unless mentioned otherwise, statistical analyses were performed within the linear mixed effects models framework. A linear mixed effects model was applied for a continuous dependent variable (e.g., certainty ratings in the recognition test) and a generalized linear mixed effects model was applied for binary dependent variables (e.g., recognition accuracy). Each model contained a random intercept across participants and centered predictors (e.g., number of options, obtained reward and SRPEs during the acquisition task). All analyses were run in R. When during the acquisition task a Swahili translation was chosen which was not framed as a possible option, the corresponding word pair was excluded from the analyses.

### Results

**Fig 2:**
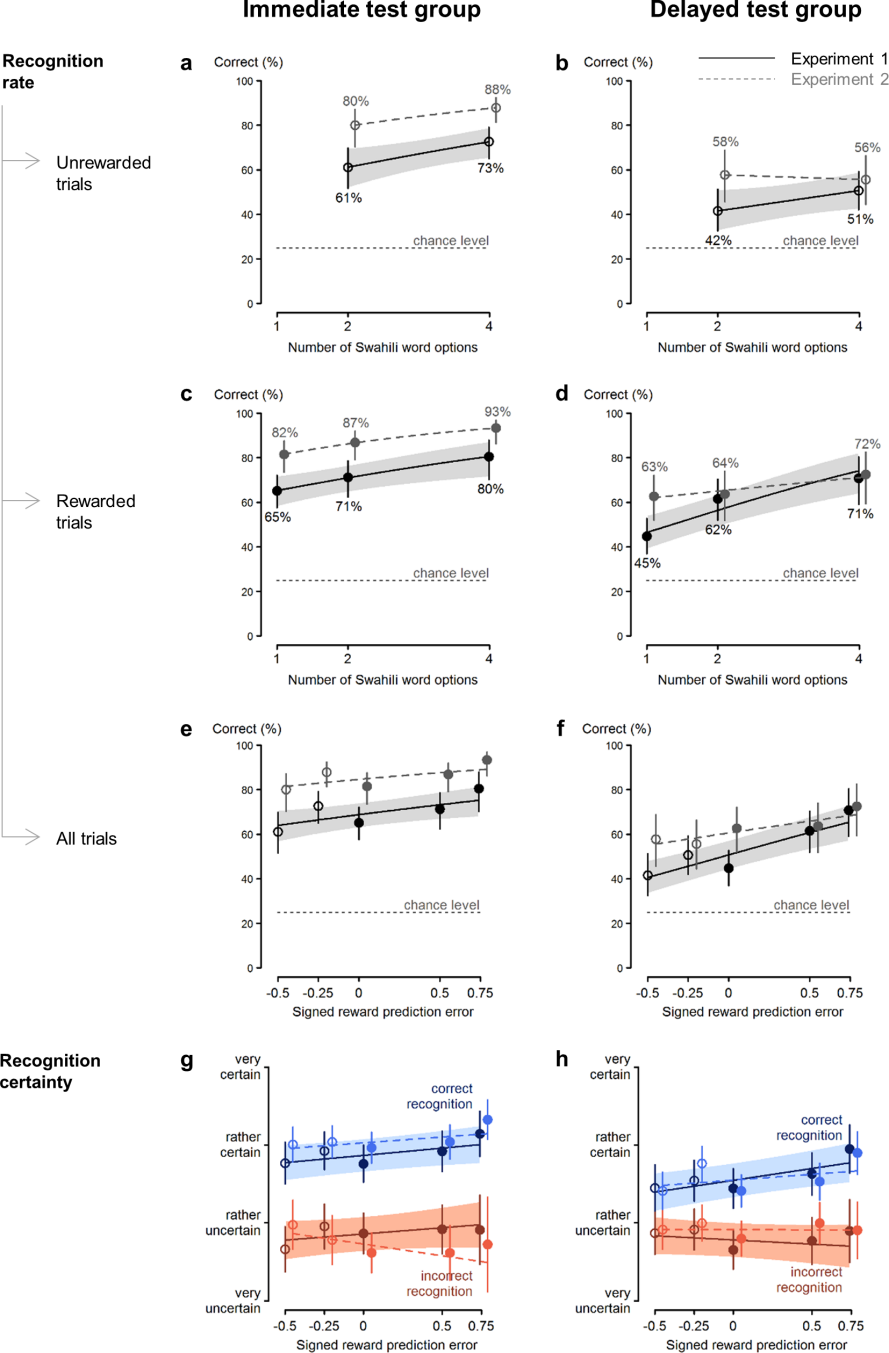
Recognition accuracy (panel a through f; y-axis) and certainty ratings (panel g and h; y-axis). Recognition accuracy and certainty ratings as a function of the number of options (panel a through d; x-axis) or the SRPEs (panel e through h; x-axis) in the immediate test group (left column) and their equivalent in the delayed test group (right column). The results of Experiment 1 are indicated by the black full line; the results of Experiment 2 are plotted with a grey dashed lines (95% confidence intervals are indicated for Experiment 1 only). To elucidate the relation between panel a-d and panel e-f, empty circles represent the unrewarded trials and full circles the rewarded trials. Note that in the one-option condition the chosen translation was always rewarded (panel a through d). For each number of options and depending on the reward and delay (as well as for the SRPEs), the average recognition accuracy/certainty and its 95% confidence interval was estimated and superimposed. (a-f) Recognition increased significantly with an increasing number of options and recognition was enhanced for rewarded word pairs; thus recognition increased significantly with higher SRPEs. Performance at chance level is indicated by the gray dotted line at 25% accuracy. (g and h) SRPEs significantly predicted certainty ratings for correctly recognized word pairs (depicted in blue) but not for incorrectly recognized word pairs (depicted in orange).

Three out of 40 participants (one in the immediate and two in the delayed test group) were removed from the dataset because of technical problems during the experiment. Mean recognition accuracies and certainty ratings per condition for Experiment 1 are presented in Fig 2 (full lines). Recognition accuracy was significantly higher in the immediate test group than in the delayed test group, χ^2^(1, *N* = 37) = 15.7, *p* < 0.001 (immediate group, 40% to 90%, *M* = 67.4%, *SD* = 14.4%; delayed group, 27% to 73%, *M* = 50.7%, *SD* = 11.6%). Therefore, test delay is included as a factor in the following analyses.

#### Differentiating the effect of URPEs and SRPEs

First, we disentangled the role of URPEs and SRPEs (Fig 1B) by testing the effect of number of options, reward, and their interaction on recognition accuracy. The URPE and SRPE accounts predict a similar pattern for positive RPEs (rewarded trials, plotted as full circles in Fig 2), but differ in their predictions for the negative RPEs (unrewarded trials; plotted as empty circles in Fig 2). That is, the URPE account predicts a significant interaction between the number of options and obtained reward, whereas the SRPE account predicts no such interaction.

As Fig 2A–2D reveals, there was a significant main effect of reward, χ^2^(1, *N* = 37) = 24.5, *p* < 0.001, with rewarded choices being remembered more accurately. In addition, recognition accuracy increased with the number of options, χ^2^(1, *N* = 37) = 36.8, *p* < 0.001. Contrary to the URPE account, but consistent with the SRPE account, there was no significant interaction between the number of options and reward, χ^2^(1, *N* = 37) = 1.42, *p* = 0.23. Note that the effects of both reward and number of options were rather large (i.e., an average accuracy increase of 13.75% across the number of options and 8.9% for reward).

As a direct test of the URPE versus SRPE accounts, we tested the number of options effect in unrewarded trials only. As mentioned above, the URPE and SRPE accounts predict an increasing and decreasing pattern with number of options, respectively. In line with the SRPE, but against the URPE account, we observed a significant increase, χ^2^(1, *N* = 37) = 9.45, *p* = 0.0021.

Furthermore, the URPE account predicts increased recognition for both large positive and large negative RPEs (depicted on abscissa in Fig 2E–2H), which would result in a quadratic effect of SRPE on memory performance. The SRPE account in contrast predicts enhanced recognition for large positive RPEs but reduced recognition for large negative RPEs, resulting in SRPE (abscissa in Fig 2E–2H) as a linear predictor of memory performance. Formally probing whether declarative memory performance improved linearly with SRPEs, recognition was significantly enhanced with increasing SRPEs, χ^2^(1, *N* = 37) = 27.4, *p* < 0.001 (Fig 2E and 2F; note that Fig 2E and 2F offers a different visualization of the same data points represented in Fig 2A–2D), consistent with the SRPE account.

As yet another way of differentiating the two models, we fitted a linear model where the SRPE terms were replaced by URPE terms (both shown in Fig 1). This URPE-based model fitted worse than the original SRPE-based one; AIC values for original (SRPE-based) and alternative (URPE-based) models were 2834.5 and 2848.7, respectively.

The certainty ratings revealed a similar pattern (Fig 2G and 2H). In line with the SRPE account, higher SRPEs resulted in significantly higher certainty ratings, χ^2^(1, *N* = 37) = 9.49, *p* = 0.0021. While the interaction between SRPE and test delay had no significant effect on recognition certainty, χ^2^(1, *N* = 37) = 0.039, *p* = 0.84, there was an interaction between SRPEs and recognition accuracy, χ^2^(1, *N* = 37) = 4.56, *p* = 0.033, and a marginally significant three-way interaction between SRPE, recognition accuracy and test delay, χ^2^(1, *N* = 37) = 3.25, *p* = 0.071. Follow-up tests revealed that SRPEs had no significant effect on certainty ratings for the false recognitions (neither in the immediate test group, χ^2^(1, *N* = 19) = 1.76, *p* = 0.18, nor the delayed test group, χ^2^(1, *N* = 18) = 2.021, *p* = 0.16), but did significantly predict certainty ratings for the correctly recognized word pairs in the immediate, χ^2^(1, *N* = 19) = 4.24, *p* = 0.039, and delayed test group, χ^2^(1, *N* = 18) = 7.27, *p* = 0.0070. The fact that the SRPEs only influence certainty ratings for the correctly recognized word pairs and not for false alarms further corroborates our finding that SRPEs drive declarative learning.

#### Testing the time-on-task account

As a first validation test, we verified whether our results could alternatively be explained by the classic time-on-task account, according to which the time spent on a task would determine recognition accuracy. To this purpose, we first tested whether longer deliberation on the one, two or four valid Swahili options on individual trials would lead to better recognition. To approximate the time devoted to each option (Swahili word) on a particular trial, we divided the deliberation time by the number of options. The resulting time-on-task per option (time-on-word) revealed that each word was examined longer when less options were available (the mean time-on-word on the one-, two- and four-option trials was 2880 ms, 1826 ms and 1169 ms, respectively). This argues against a time-on-task account as recognition performance increased with the number of options while the time-on-word decreased when more options were available. We then tested whether increased (trial-to-trial) time-on-word would improve recognition. Counter to the predictions from the time-on-task account, there was no significant influence of time-on-word on recognition, χ^2^(1, *N* = 37) = 1.48, *p* = 0.22. Follow-up tests for one-, two- or four-option trials separately confirmed that recognition was not significantly influenced by the (trial-to-trial) time-on-word (one-option trials, χ^2^(1, *N* = 37) = 0.096, *p* = 0.76; two-option trials, χ^2^(1, *N* = 37) = 0.026, *p* = 0.87; four-option trials, χ^2^(1, *N* = 37) = 2.52, *p* = 0.11). The result of the one-option trials is particularly interesting as participants could already start learning the word pair during the deliberation time. Still, even in the one-option condition longer deliberation on the valid Dutch-Swahili word pair failed to result in better declarative learning.

## Experiment 2

Experiment 1 demonstrated a clear effect of SRPE on declarative memory. As a second validation test and replication of this finding, in Experiment 2 we investigated the generalizability across input modalities. Here we tested the effect of RPE on the acquisition of pictures rather than words. The experimental design is the same as in Experiment 1 unless noted otherwise (i.e., the design was slightly adjusted to better fit future EEG research; no EEG data were currently collected).

### Methods

#### Participants

Forty participants (29 female) were randomly assigned to either the immediate or delayed test group (20 participants in each group). None of the participants had previously taken part in Experiment 1 or had any knowledge of Swahili.

#### Materials

A total of 240 Swahili words were used (identical to Experiment 1, see Table 1 and Table 2) and 60 figures were selected from the colorized Snodgrass and Vanderwart dataset [20,21]. Like Experiment 1, Experiment 2 consisted of three parts: the familiarization task, the acquisition task and the recognition test.

#### Familiarization task

Participants were shown the 240 Swahili words, randomly intermixed with 60 figures accompanied by their Dutch meaning. The stimuli appeared in random order for a duration of two seconds each. Participants were instructed to press the spacebar whenever a figure was shown.

#### Acquisition task

At the beginning of the acquisition task, participants were told they would learn 60 figure-word pairs and would earn up to €10 for taking part in the study. They were reminded that a gift voucher of €20 would be awarded to the participant with the best recognition performance.

**Fig 3:**
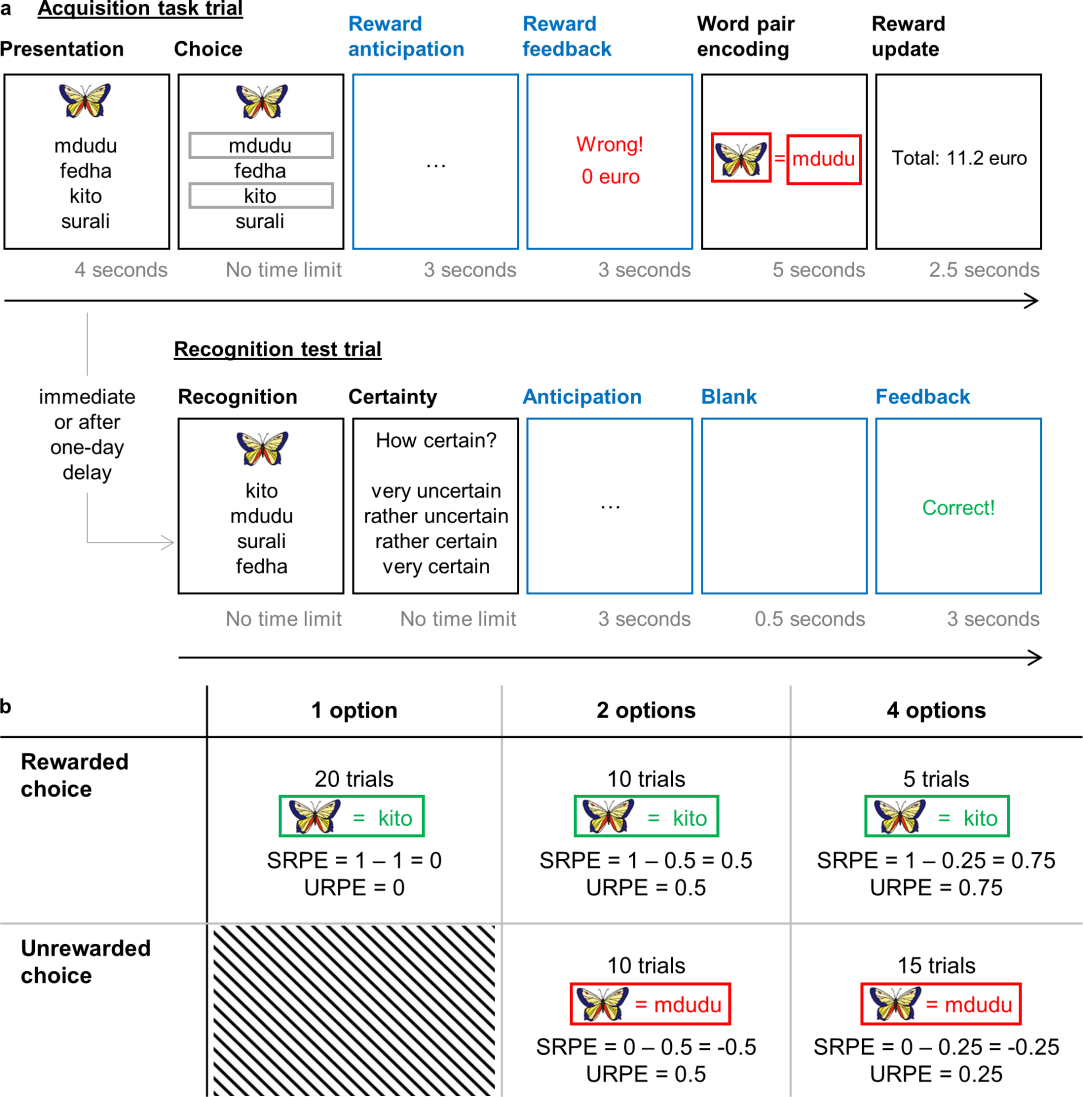
**Experiment overview (a) and experimental design (b) for Experiment 2.** The general trial structure and experimental design of Experiment 2 are largely a replication of Experiment 1.

On each trial, one figure was accompanied by four Swahili words (Fig 3A). After four seconds, frames surrounded either one, two or four Swahili words. These frames indicated out of which Swahili translations participants were allowed to choose as the translation for the figure (no time constraint). After participants made their choice, there was a reward anticipation phase (three seconds). Participants were then given reward and performance feedback (three seconds) followed by the to-be-learned figure-word pair (five seconds). Each trial ended with a 2.5 seconds reward update indicating how much participants had earned up until the last completed trial. Note that the reward schedule of Experiment 2 is exactly the same as in Experiment 1 (Fig 3B), thus all participants were rewarded €9.80 (rounded to €10) for a total of 35 rewarded trials.

#### Recognition test

The magnitude comparison task was again presented as a filler task to reduce recency effects in the recognition test.

On each trial of the recognition test, one figure was presented at the top of the screen together with the same four Swahili words as in the acquisition task (Fig 3A). In analogy to Experiment 1, the order of the Swahili words was randomized to avoid learning based on spatial position. No time constraint was imposed. After selecting their response, participants were asked how certain they were of their answer. At the end of each trial, they were given feedback on the accuracy of their answer.

### Results

The results of Experiment 2 largely replicated those of Experiment 1, and will therefore be reported more concisely. Accuracy was significantly higher in the immediate test group compared to the delayed test group, χ^2^(1, *N* = 40) = 19.1, *p* < 0.001 (immediate group, 55% to 100%, *M* = 81.5%, *SD* = 13.5%; delayed group, 37% to 93%, *M* = 59.3%, *SD* = 16.2%).

The data revealed a significant main effect of reward, χ^2^(1, *N* = 40) = 13.3, *p* < 0.001 (Fig 2A–2D, dashed grey lines depict results from Experiment 2). Recognition accuracy was higher for rewarded choices compared to unrewarded choices. Recognition accuracy also increased with number of options, χ^2^(1, *N* = 40) = 10.2, *p* = 0.0014. Importantly, the interaction between reward and number of options was not significant, χ^2^(1, *N* = 40) = 2.17, *p* = 0.14. These results are again in favor of the SPRE account. Effects of both reward and number of options were again rather large (i.e., an average accuracy increase of 4.75% across the number of options and 6.33% for reward).

Next, we verified whether recognition accuracy linearly increased with SRPEs. There was a significant positive effect of SRPE, χ^2^(1, *N* = 40) = 13.4, *p* < 0.001, with larger and more positive RPEs leading to increased recognition accuracy (Fig 2E–2F). Then, as in Experiment 1, we fitted an alternative URPE-based model to compare it with the SRPE-based one. As in Experiment 1, the AIC value was lower (better fit) for the SRPE-based model (AIC values are 2596 and 2588 for URPE-based and SRPE-based models, respectively).

For the certainty ratings there was a significant main effect of recognition accuracy, χ^2^(1, *N* = 40) = 426, *p* < 0.001, indicating that participants were more certain of correctly recognized figure-word pairs (Fig 2G and 2H). In addition, the certainty ratings revealed a significant interaction between SRPE and recognition accuracy, χ^2^(1, *N* = 40) = 5.32, *p* = 0.021. Follow-up analysis showed that SRPE only influenced certainty for correctly recognized figure-word pairs, χ^2^(1, *N* = 40) = 6.90, *p* = 0.0086, but not for incorrectly recognized figure-word pairs, χ^2^(1, *N* = 40) = 0.97, *p* = 0.33. In line with Experiment 1, SRPE thus only increased certainty for correctly recognized figure-word pairs and had no effects on false recognitions.

Finally, the time-on-word analysis resulted in the same pattern of results as in Experiment 1. The time-on-word decreased as the number of options increased (2762 ms, 1770 ms and 1007 ms, for the one-, two- and four-option trials respectively) and failed to significantly predict the recognition accuracy, χ^2^(1, *N* = 40) = 0.058, *p* = 0.81.

## Discussion

In two experiments, we demonstrate that signed reward prediction errors (SRPEs) drive declarative learning. Earlier work already demonstrated effects of reward and RPEs on perceptual, procedural, and motor learning; of reward on declarative learning; of RPE on neural responses in declarative learning; and of RPEs on recognition criterion setting. However, in the current study we provide direct empirical evidence on whether RPEs influences performance in declarative learning. To do so, positive and negative RPEs of known and various sizes were generated by manipulating the number of options available in a vocabulary acquisition task. Perhaps the most striking aspect of our findings was that more response options improved performance, which is predicted by our SRPE account, but against intuition (or the time-on-task account) because subjects can actually start studying more quickly when there are fewer alternatives. Thus, our results provide the first demonstration that stimuli associated with large, positive RPEs during learning, are later recognized more accurately and with higher certainty; despite only a single exposure during declarative learning. In addition, while the importance of URPEs (“different than expected” signals [22,23]) has been shown in procedural learning paradigms [5,11,24] our analysis suggests that declarative learning is driven by SRPEs (“better than expected” signals).

These results further our understanding of how motivational cues determine which information is prioritized for encoding in memory. As discussed previously, the neoHebbian learning account [10] predicts that declarative learning depends on pre- and postsynaptic activity, which relation is further modulated by dopamine bursts. These dopaminergic responses are thought to follow an SRPE signature, with a stronger response to outcomes that are better than expected [7]. Critically, these dopamine bursts can be caused by a variety of motivational cues such as RPEs, novelty and salience. Previous research has indeed demonstrated that declarative learning is enhanced by reward anticipation [12,13], exposure to novel environments [25], exposure to prediction errors not related to reward [26] and the exposure to salient (emotional) stimuli more generally [27]. Critically, we provide a first empirical validation of the effect of RPEs on (behavioral performance in) declarative memory (in humans).

Beyond the neoHebbian account, our findings resonate with a recent interest in relationships and overlaps between concepts developed in the declarative and procedural learning literatures (e.g., [28,29]). Metcalfe reviews a body of work demonstrating the important role of making errors in declarative memory performance [30]. For example, Metcalfe and colleagues have extensively reported on the hypercorrection effect, showing that high-confidence errors are easier to correct than low-confidence ones (e.g., [31]). Tricomi and colleagues have in several papers shown that caudate nucleus (typically associated with procedural learning) is also activated in feedback processing in declarative learning. An important finding emerging from this work is that caudate is not just active when reward (either in declarative or procedural contexts) is processed; but that caudate is activated to the extent that the feedback is useful for learning about task contingencies. This is explained based on a goal attainment theory of caudate nucleus (e.g., [32–34]). It is not clear at this time how neoHebbian, error, and goal attainment theories can be conceptually integrated. However, what is clear is that a rich set of interactions between different types of learning, usually studied separately, remain to be explored, at both theoretical and empirical levels.

As declarative learning plays a predominant part in education, these results stress the need for a better understanding of the role that reward (and its prediction) plays in declarative learning. Despite an early reluctance of educational theorists toward incorporating reward in educational settings, its role has been reconsidered in recent years [35]. One potential illustration is the testing effect [36]. The testing effect refers to the finding that testing, rather than mere studying, dramatically improves performance in a later recall test [37,38]. In a seminal publication, Karpicke and Roediger empirically manipulated the amount of study and test trials allotted to Swahili-English word pairs [38]. In a follow-up test one week later, the authors found that additional study trials during the acquisition session had no strong beneficial effect on retention. Conversely, recall was strongly enhanced by additional test trials during acquisition. Although this testing effect has consistently been observed to drive declarative learning and holds major educational implications [39], its origin has remained unclear. From the current standpoint, we may reinterpret this finding as resulting from RPEs. In particular, we hypothesize that during testing, predictions are generated that are then followed by either external feedback (from an instructor or experimenter) or by internal self-generated feedback. Internal and external feedback indeed have the same neural signatures [40]. Such feedback may generate RPEs, resulting in a facilitatory effect of testing. Thus, active predictions and their entailing RPEs may drive declarative learning (even in the absence of external feedback [41,42]). An interesting case in point is a study in which participants learned cue-target word pairs with a strong or weak semantic association [43]. Whereas restudying the material equally improved the retention of strongly and weakly associated word pairs, repeated testing improved recall of weakly associated word pairs more compared to strongly associated words. Moreover, in the final test the recall for the weak semantic associations surpassed that of the strong semantic associations. Although counter-intuitive at first glance, these findings follow naturally from the beneficial effect of RPEs on declarative learning as weak associations leave more room for the formation of large RPEs. More broadly, the natural occurrence of RPEs during learning might be why testing, elaborative interrogation and self-explanation outperform other active learning strategies such as summarizing, keyword mnemonics and imagery [44]. Future research should make this connection more direct, especially in light of the recent trend toward gamification in educational settings.

In sum, we demonstrate that SRPEs drive declarative learning, closing the gap between research on reward learning and declarative memory. Our results are in line with the neoHebbian learning framework and suggest new avenues to improve learning in both informal and educational settings.
